# Retraction: Inhibition of Bcl2L12 attenuates eosinophilia-related inflammation in heart

**DOI:** 10.3389/fimmu.2023.1227707

**Published:** 2023-06-05

**Authors:** Frontiers Editorial Office

Following publication, concerns were raised regarding the validity of the data in the article. The authors failed to provide a satisfactory explanation during the investigation, which was conducted in accordance with Frontiers’ policies. Given the concerns the editors no longer have confidence in the findings presented in the article.

This retraction was approved by the Chief Editors of Frontiers in Immunology- Inflammation and the Chief Executive Editor of Frontiers. The authors agree to respond to correspondence regarding this retraction.

